# Small Molecule NF-κB Inhibitors as Immune Potentiators for Enhancement of Vaccine Adjuvants

**DOI:** 10.3389/fimmu.2020.511513

**Published:** 2020-09-25

**Authors:** Brittany A. Moser, Yoseline Escalante-Buendia, Rachel C. Steinhardt, Matthew G. Rosenberger, Britteny J. Cassaidy, Nihesh Naorem, Alfred C. Chon, Minh H. Nguyen, Ngoctran T. Tran, Aaron P. Esser-Kahn

**Affiliations:** ^1^Pritzker School of Molecular Engineering, The University of Chicago, Chicago, IL, United States; ^2^Department of Chemistry, Chemical Engineering and Materials Science, Biomedical Engineering, University of California, Irvine, Irvine, CA, United States

**Keywords:** adjuvants, vaccines, NF-κB, honokiol, capsaicin

## Abstract

Adjuvants are added to vaccines to enhance the immune response and provide increased protection against disease. In the last decade, hundreds of synthetic immune adjuvants have been created, but many induce undesirable levels of proinflammatory cytokines including TNF-α and IL-6. Here we present small molecule NF-κB inhibitors that can be used in combination with an immune adjuvant to both decrease markers associated with poor tolerability and improve the protective response of vaccination. Additionally, we synthesize a library of honokiol derivatives identifying several promising candidates for use in vaccine formulations.

## Introduction

Vaccines remain one of the most effective ways of preventing disease. Despite their immense success in preventing diseases such as polio, tetanus, and small pox, diseases such as HIV and dengue present challenges that current clinical vaccine technologies cannot provide. To solve this problem, one strategy that has been explored is to include adjuvants, molecules that enhance the immune response (1). Although novel adjuvants generate higher quality immune responses that cannot be achieved with current approved adjuvants, to date, very few have been approved for use in human vaccines. This disconnect is due, in part, to the challenge of balancing the proinflammatory immune response with the protective, adaptive immune response (2–4). We recently reported that vaccines could be improved through the use of a peptide NF-κB inhibitor, SN50 in combination with an immune adjuvant (5). The addition of SN50 to adjuvanted vaccines led to increased safety of the adjuvant while enhancing protection against disease. Although this method proved both general across a wide range of adjuvants and effective against antigens of a variety of diseases, it still required a large amount of the peptide to enable optimal safety and protection. Scale-up of peptides present synthetic challenges and can result in expensive production costs, limiting their potential in a clinical setting (6, 7). Peptides might also induce an immune response against themselves leading to a potential for decreased enhancement in subsequent vaccinations. We chose to explore other small molecule NF-κB inhibitors as immune potentiators to overcome these challenges.

Here we demonstrate that select small molecule NF-κB inhibitors are effective at reducing adjuvant-induced inflammation while also increasing the adaptive humoral immune response. At the same time, we demonstrate that not all NF-κB inhibitors are effective immune potentiators. Of the molecules we tested, honokiol and capsaicin proved to be effective at both limiting inflammation and potentiating the protective response. Through knockout studies, we demonstrate that the increase in antigen specific antibodies is independent from the anti-inflammatory activity, which is congruent with our previous studies (5). To determine if these small molecules could be improved by chemical synthesis, we explored derivatives of honokiol and found several promising candidates for potential use in vaccines.

## Results and Discussion

### Exploration of Small Molecule NF-κB Inhibitors *in vitro*

**FIGURE 1:**
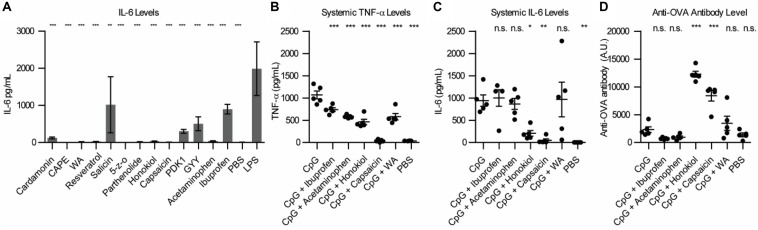
Small molecule inhibitor screen *in vitro* and *in vivo*. **(A)** IL-6 levels from RAW macrophages 24 h post-stimulation with NF-κB inhibitor and LPS. Significance is compared to LPS alone, *n* = 3. **(B)** Systemic TNF-α expression 1 h post-vaccination, *n* = 5. **(C)** Systemic IL-6 expression 1 h post-vaccination, *n* = 5. **(D)** Anti-OVA antibody level 21 days post-vaccination, *n* = 5. Significance is compared to CpG vaccination. *p < 0.05, **p < 0.01, ***p < 0.001.

To begin exploring alternative NF-κB inhibitors, we examined the literature for promising candidates. Due to the strong correlation between NF-κB activation and sepsis (8), cancer (9, 10) and autoimmune disorders (11), a large library of NF-κB inhibitors have been identified (12). Small molecule NF-κB inhibitors often do not act on the NF-κB subunits themselves, but rather inhibit particular proteins in the NF-κB pathway, upstream of NF-kB translocation to the nucleus (see Supplementary Table S1). We first wanted to analyze the potential of a variety small molecule NF-κB inhibitors to inhibit inflammation *in vitro* in combination with lipopolysaccharide (LPS), a TLR4 agonist. We chose several common commercially available NF-κB inhibitors and tested them in RAW macrophages. We chose to examine: Cardamonin (40 μM), Caffeic acid phenethyl ester (CAPE) (100 μM), Withaferin A (WA) (400 nM), Resveratrol (10 μM), Salicin (100 nM), 5Z-7-Oxozeaenol (5-z-O) (5 μM), Parthenolide (20 μM), Honokiol (20 μM), Capsaicin (200 μM), PDK1/Akt/Flt dual pathway inhibitor (PDK1) (1 μM), and GYY 4137 (GYY) (200 μM). To determine if immune potentiation was specific to NF-κB or general to all anti-inflammatory molecules, we included the most common FDA approved anti-inflammatory drugs, acetaminophen (10 mM) and ibuprofen (800 μM) (13, 14). We treated RAW macrophages with inhibitors and LPS and assayed the supernatant for IL-6 secretion (Figure 1A). CAPE, WA, 5-z-O, honokiol and capsaicin demonstrated the greatest reduction in IL-6 levels.

### Exploration of Small Molecule NF-κB Inhibitors *in vivo*

We next wanted to examine how these inhibitors would alter safety and protection *in vivo*. To test this *in vivo*, we chose three of the small molecule inhibitors that were the most effective at inhibiting IL-6 expression *in vitro*, capsaicin, honokiol and withaferin A (WA) and ran them alongside acetaminophen and ibuprofen. We chose to vaccinate mice using CpG, a TLR9 agonist. For our *in vivo* vaccination, we used ovalbumin (OVA) as a model antigen to examine the changes in humoral response. We vaccinated mice with 100 μg OVA, 50 μg CpG, and inhibitor (800 μg ibuprofen, 2 mg acetaminophen, 400 μg honokiol, 20 μg capsaicin or 600 μg WA). Due to the difficulty in solubility of the inhibitors, all vaccines were formulated in Addavax, a squalene-based oil-in-water nano-emulsion, to enable effective vaccine suspensions. To enable comparison between groups PBS and CpG controls were also formulated in Addavax. We chose to analyze systemic levels of TNF-α and IL-6 because high levels of these cytokines are pyrogenic and have been correlated with undesirable vaccine-related side effects (15–17). We previously determined that CpG-induced TNF-α and IL-6 peak at 1 h post-vaccination (5). Mice vaccinated with CpG demonstrated high levels of TNF-α (1067 pg/mL) (Figure 1B). Addition of an NF-κB inhibitor decreased the level of TNF-α. Ibuprofen decreased to the mean level of TNF-α to 738 pg/mL (1.4 fold), acetaminophen 584 pg/mL (1.8 fold), honokiol 464 pg/mL (2.3 fold), capsaicin 38 pg/mL (28 fold, equivalent to background levels), and WA 580 pg/mL (1.8 fold). The systemic levels of IL-6 were also high with CpG vaccination (941 pg/mL). The groups that included an NF-κB inhibitor did not always decrease the level of IL-6 (Figure 1C). Ibuprofen, acetaminophen and WA did not alter the cytokine profile statistically significantly compared to CpG alone. Ibuprofen (1001 pg/mL) increased the level of IL-6 by 1.06 fold. Acetaminophen (866 pg/mL) decreased the level by 1.08 fold. WA increased the level of IL-6 to 967 pg/mL (1.02 fold increase). However, honokiol and capsaicin dramatically reduced the systemic levels of IL-6 to 206 pg/mL (3.5 fold) and 47 pg/mL (20 fold), respectively.

To broadly establish how the addition of these inhibitors impacts the antibody levels, we chose to analyze the total Ig (G+A+M) produced after 21 days (18). On day 21, we analyzed the anti-OVA antibody levels (Figure 1D). CpG was 1.6 fold (2312 U/mL) higher than PBS (1365 U/mL). Ibuprofen (708 U/mL) and acetaminophen (955 U/mL) were 3.2 and 2.4 fold lower that CpG alone. CpG + honokiol (12286 U/mL) was 5.3 fold more than CpG alone. CpG + capsaicin (8413 U/mL) was 3.6 fold higher than CpG alone. CpG + WA (3459 U/mL) was 1.5 fold higher than CpG alone.

These results demonstrate that honokiol and capsaicin are capable of both mitigating the systemic proinflammatory cytokines, TNF-α and IL-6, while also increasing the adaptive humoral response. WA demonstrated a decrease of systemic TNF-α while maintaining a similar antibody level as CpG alone. We were unable to formulate vaccines using CAPE and 5-z-O due to solubility issues; however, we believe they are worth exploring in future studies using alternative formulations.

### Dose-Dependence of Capsaicin and Honokiol

**FIGURE 2:**
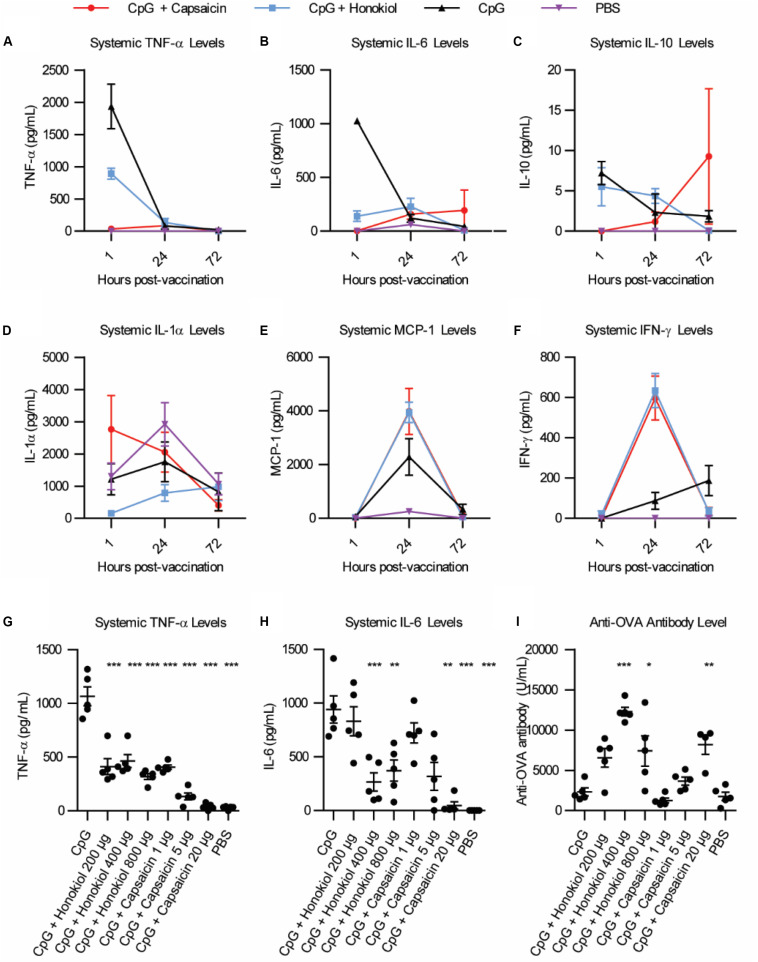
Broader cytokine response and dose effects of honokiol and capsaicin. **(A–F)** Systemic cytokine levels at 1, 24, and 48 h post-vaccination, *n* = 5. CpG (black line), CpG + Capsaicin (red line), CpG + Honokiol (blue line), PBS (purple line) **(G)** Systemic TNF-α levels 1, 24, and 72 h post-vaccination with varying doses of honokiol and capsaicin, *n* = 5. **(H)** Systemic IL-6 levels 1 h post-vaccination, *n* = 5. **(I)** Anti-OVA antibody levels 21 days post-vaccination, *n* = 5. Significance is compared to CpG alone. *p < 0.05, **p < 0.01, ***p < 0.001.

Of the candidates, Capsaicin and honokiol demonstrated exceptional promise in these studies so we examined them further. To better understand how these molecules are altering the immune response over time, we vaccinated mice as described above and analyzed a larger variety of cytokines at various timepoints. We analyzed 13 cytokines: IL-1α, IL-1β, IL-6, IL-10, IL-12p70, IL-17A, IL-23, IL-27, MCP-1, IFN-β, IFN-γ, TNF-α, and GM-CSF. Of these, only six cytokines demonstrated detectable levels in our assay: TNF-α, IL-6, IL-10, IL-1α, MCP-1, and IFN-γ (Figures 2A–F). Consistent with our previous findings (5), CpG induced TNF-α and IL-6 expression peaked at 1 h. Interestingly, CpG combined with either capsaicin or honokiol had increased IFN-γ levels at 24 h compared to CpG alone (8 fold and 9 fold, respectively) and slightly elevated MCP-1 levels (2.5 and 2 fold, respectively), demonstrating that both capsaicin and honokiol are acting to potentiate the immune response and are not simply suppressing immune activation. We next wanted to understand how changing the dose would alter innate and adaptive humoral immune responses. For honokiol, we tested a concentration 2-fold higher (800 μg) and 2-fold lower (200 μg) than the original dose (400 μg). Mice vaccinated with our original dose of capsaicin (20 μg) appeared lethargic for 30 min post-injection, therefore we wanted to examine if we could lower the dose, but maintain adequate anti-inflammatory activity and antibody-boosting potential. We chose to test a dose 4-fold lower (5 μg) and 20-fold lower (1 μg) than the original dose (20 μg). Unfortunately, mice vaccinated with all doses of capsaicin appeared lethargic post-injection, however, mice vaccinated with the lowest dose of capsaicin (1 μg) only appeared lethargic for ten minutes. All doses of honokiol demonstrated a significant decrease in TNF-α expression compared to CpG alone, however, there was no significant difference between the different doses (Figure 2G). Capsaicin decreased TNF-α levels significantly across all doses compared to CpG alone. Capsaicin doses of 5 and 20 μg decreased levels of TNF-α significantly more than 1 μg (Figure 2G). The level of IL-6 was only decreased with 400 μg and 800 μg honokiol and 20 μg capsaicin (Figure 2H). Twenty-one days later, we analyzed differences in anti-OVA antibody level and found that all doses of honokiol increased levels of anti-OVA antibodies compared to CpG alone and the highest level was found with 400 μg honokiol (Figure 2I). 1 μg and 5 μg of capsaicin did not change level of anti-OVA antibodies in the serum compared to CpG alone, however, 20 μg significantly increased serum levels. These results indicate that honokiol can only limit TNF-α and IL-6 to a certain extent at which higher doses do not provide additional decreases in these systemic inflammatory cytokines. Additionally, the highest dose of honokiol decreased the antibody level, indicating that there is an optimal dose at which this inhibitor can function as an immune potentiator. Capsaicin demonstrated a dose-dependent response, where higher doses of capsaicin led to lower systemic TNF-α and IL-6 levels and higher antibody levels. This would point to a very promising candidate as an immune potentiator, however, these mice experienced other undesirable side effects (lethargy).

### Determining the TRPV1-Mediated Effects of Capsaicin

**FIGURE 3:**
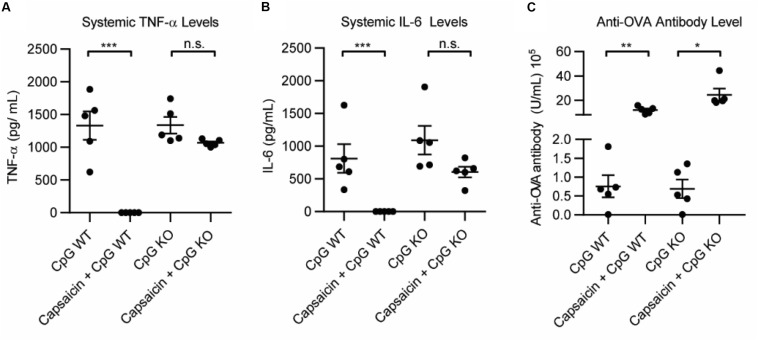
Role of TRPV1 of capsaicin induced anti-inflammatory and immune potentiation. **(A)** Systemic TNF-α levels 1 h post vaccination in wild type (WT) mice and TRPV1 KO (KO), *n* = 5. **(B)** Systemic IL-6 levels 1 h post-vaccination, *n* = 5. **(C)** Anti-OVA antibody level 21 days post-vaccination, *n* = 5. *p < 0.05, **p < 0.01, ***p < 0.001.

The primary *in vivo* target for capsaicin is the transient receptor potential cation channel subfamily V member 1 (TRPV1). TRPV1 modulates the immune response in a variety of ways, and importantly, has been implicated in dampening systemic inflammation associated with sepsis (19–23). However, it has never been explored in a vaccine setting. To understand how activation of TRPV1 may be modulating the effects of the adjuvant, we compared the immediate inflammatory response of the vaccination in wild type mice (WT) and TRPV1 knockout mice. We vaccinated WT and TRPV1 KO mice with 100 μg OVA and: 50 μg CpG, 50 μg CpG + 20 μg capsaicin or PBS. We analyzed systemic levels of TNF-α and IL-6 1 h after vaccination. We found that CpG induced high levels of TNF-α and IL-6 in both WT and TRPV1 KO mice. Addition of capsaicin dramatically and significantly reduced both TNF-α levels and IL-6 levels in the WT mice (Figures 3A,B and Supplementary Figure S1). Although the mean was slightly lower for both TNF-α and IL-6 in the TRPV1 KO mice, these differences were not statistically significant. This demonstrated that TRPV1 activation is responsible for the capsaicin-induced decrease in systemic cytokine levels. As expected, the TRPV1 KO mice did not experience the lethargy experienced by the WT mice in response to capsaicin, indicating that activation of TRPV1 is responsible for this response. To examine if the increased antibody level was due to TRPV1 activation on day 21, we analyzed levels of anti-OVA antibodies in the serum (Figure 3C and Supplementary Figure S1). Interestingly, we found that anti-OVA antibody levels were increased in groups with Capsaicin + CpG in both WT and KO mice. This implies that the antibody-boosting activity of capsaicin is separate from TRPV1-dependent decrease in inflammatory cytokines. This result demonstrates both that the decrease in inflammation is not responsible for the antibody-boosting activity of the NF-κB inhibitor and also that the enhancement of the adaptive humoral response is independent of TRPV1 activation. These results, while not definitive, showed two separate, but correlated mechanisms for capsaicin that result in the reduction in cytokines and increase antibody levels. As such, capsaicin did not warrant further examination as a potential clinical immune potentiator. We will explore the mechanistic implications of this for immune potentiators more broadly in future publications.

### Synthesis of Honokiol Derivative Library

**SCHEME 1:**
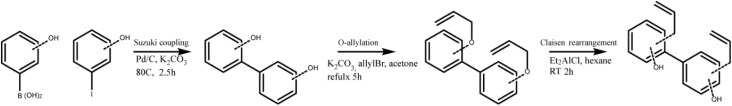
Honokiol derivative synthesis.

**FIGURE 4:**
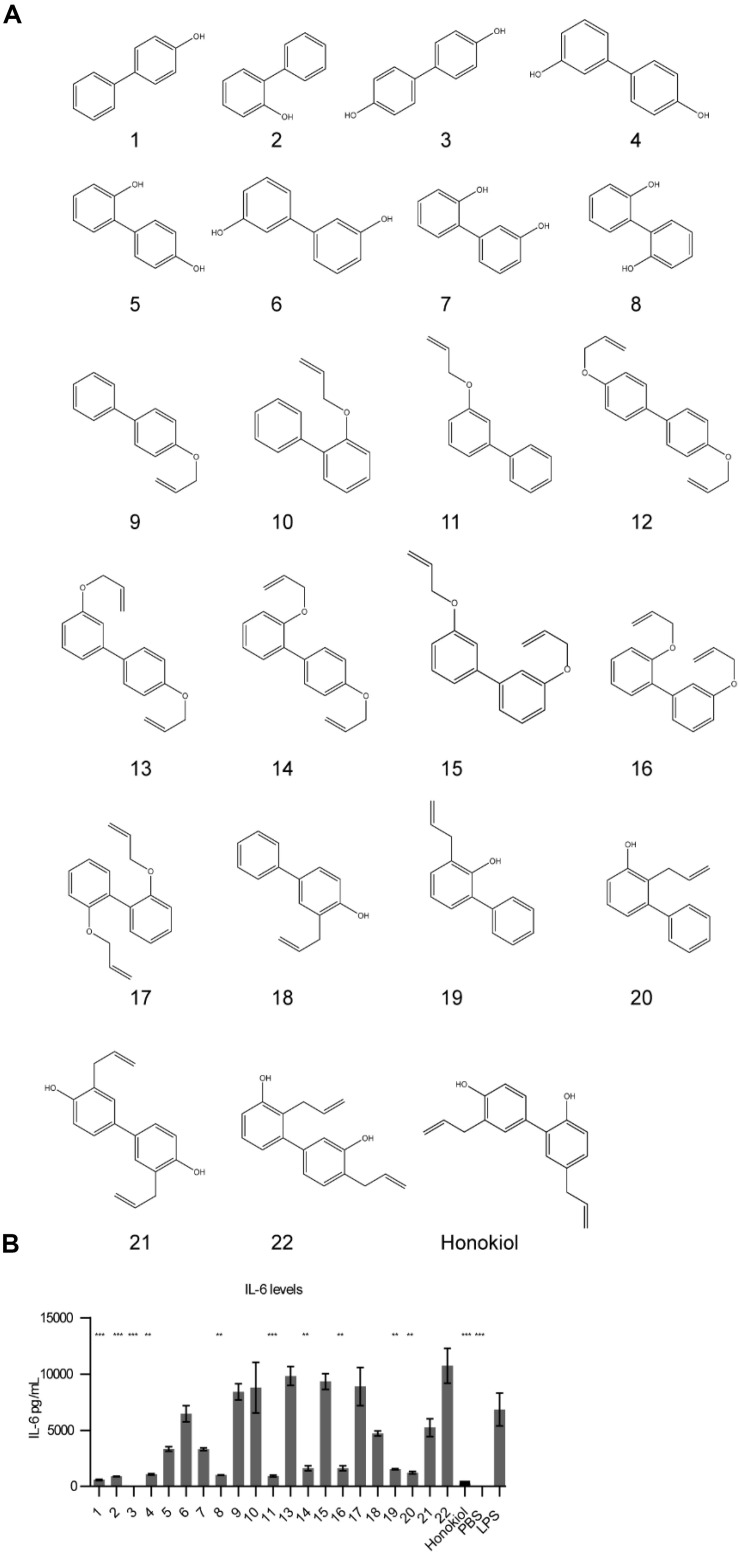
Honokiol derivatives and their inhibitory activity on IL-6 expression. **(A)** Honokiol derivative library **(B)** IL-6 expression of RAW macrophages treated with honokiol derivatives and LPS, *n* = 3. Significance is compared to LPS alone. **p < 0.01, ***p < 0.001.

With capsaicin possessing two parallel mechanisms and possessing well-established side effects (24), we wanted to explore honokiol for further development as a candidate. Oral intake of honokiol has been well studied in humans and has been established as safe with little to no side effects (25). However, it is subject to glucuronidation, leading to fast clearance (25). Additionally, vaccines for various diseases may require diverse potentiator activities. An important question for immune potentiators and honokiol was if standard SAR methods would yield alteration in potentiation activity. We wanted to investigate if compound libraries of promising immune potentiator candidates could be synthesized and provide alterations to activity. To further explore this idea, we synthesized a library of honokiol derivatives. Honokiol derivative libraries have been synthesized previously and examined for their effects on neuroprotection (26), antimicrobial agents (27) and anti-cancer (28) among others (29, 30). However, to date no such study has examined the effects of honokiol analogs on vaccines or a combination of anti-inflammatory activity and adaptive humoral immune response. Phenylphenols and biphenols were prepared using Pd-catalyzed Suzuki coupling using corresponding iodophenols and hydroxyphenylboronic acids as starting materials. These compounds were *O*-allylated using allylBr. Resulting compounds were subjected to Claisen rearrangement using diethyl aluminum chloride to yield a variety of ring substitutions (Scheme 1 and Figure 4A).

We analyzed how the honokiol derivatives altered IL-6 production in RAW macrophages. We chose to analyze the hydroxybiphenyls and *O*-allylated derivatives in addition to the product from the Claisen rearrangement to understand how these functional groups play a role in the anti-inflammatory action or increase in adaptive immune response (Figure 4B and Supplementary Figures S2, S3). We treated RAW macrophages with honokiol derivatives and LPS and analyzed IL-6 expression. The addition of LPS alone without a honokiol derivative gave high levels of IL-6 expression (6848 pg/mL). The addition of honokiol decreased IL-6 levels to 260 pg/mL, a decrease of 26-fold. Several derivatives including compounds: **1**, **2**, **3**, **4**, **8**, and **11** demonstrated similar reductions in IL-6 expression *in vitro* making them promising candidates for *in vivo* analysis. As a small molecule, honokiol impacts multiple pathways aside from NF-κB such as: STAT3, EGFR, mTOR, and caspase-mediated common pathways (31). It is probable that alterations to the structure will impact which pathways are modulated, altering the downstream response. It is likely that different vaccine formulations will require potentiators with unique modulatory characteristics as well as pharmacokinetic/pharmacodynamic properties. Compounds lacking a free hydroxyl group (such as **11**) are unable to form glucuronide conjugates, potentially extending the half-life. Here we demonstrated that compound libraries can be synthesized and screened in vitro for immune activity and that alterations to the structure change the downstream response. Such libraries could be used for improving rational design of future immune potentiators through machine learning. In future studies we will explore the effects of these inhibitors *in vivo*.

## Conclusion

In summary, we present that select small molecule inhibitors of NF-κB can decrease the inflammatory effects of adjuvanted vaccination – potentially enabling safer vaccination while also acting as immune potentiators and increasing the antibody level. We identified two such immune potentiators, honokiol and capsaicin that effectively decrease inflammation while increasing the adaptive humoral response. We additionally provide evidence that implies that the decrease in inflammation is separate from the increase in antibody response, potentially enabling distinct tunability of either response. This study also identifies that only select NF-κB inhibitors can be used as immune potentiators, this broadens the potential for further modulation of the immune response. We additionally synthesized and examined a library of honokiol derivatives and found that several honokiol derivatives are promising candidates for future testing *in vivo*. In the future, this information can be used in modern screening methods involving machine learning to identify better immune potentiators. The use of combining NF-kB inhibitors with vaccine adjuvants could find use in creating next-generation prophylactic vaccines and immunotherapy applications. In conclusion, we have demonstrated that using small molecule NF-κB inhibitors in combination with common immune adjuvants can decrease the production of pro-inflammatory cytokines TNF-α and IL-6 while boosting antibody levels.

## Materials and Methods

### *In vitro* Assays

#### RAW Macrophage Cytokine Analysis

RAW 264.7 macrophages were passaged and plated in a cell culture treated 12- well plate at 0.5 × 10^6^ cells/well in 1 mL DMEM containing 10% FBS. Cells were grown for 2 days. Media was exchanged for 1 mL DMEM containing 10% HIFBS. Inhibitors were diluted in Addavax and then in PBS. Inhibitors were added at indicated concentrations and incubated for 45 min. After 45 min, LPS was added at 100 ng/mL and incubated at 37°C and 5% CO_2_ for 24 h. Cell supernatant was removed and analyzed using BD Cytometric Bead Array Mouse Inflammation Kit.

#### Cell Viability Assay

RAW macrophages were plated at 100 k cells/well in 180 uL DMEM/10% HIFBS. Inhibitors were diluted as described above and added at indicated concentrations and incubated for 45 min. After 45 min, LPS was added to a final concentration of 100 ng/mL and incubated at 37°C and 5% CO_2_ for 24 h. MTT reagent was made fresh at a concentration of 5 mg/mL in PBS and sterile filtered. 150 μL cell supernatant was removed and 150 μL PBS was added. 10 μL MTT reagent was added to each well and incubated at 37°C and 5% CO_2_ for 2 h. 150 μL supernatant was removed from each well and replaced with 150 μL DMSO and incubated at 37°C and 5% CO_2_ for 1 h or until purple crystals dissolved. Plate was analyzed using Multiskan FC plate reader (Thermo Fisher Scientific) and absorbance was measured at 450 nm. Data was analyzed using Graphpad Prism.

#### Flow Cytometry

RAW macrophages (2 × 10^6^) were plated in a 12 well plate in DMEM/10% HIFBS. Inhibitors were diluted as described above and added at indicated concentrations and incubated for 45 min. After 45 min, LPS was added to a final concentration of 100 ng/mL and incubated at 37°C and 5% CO_2_ for 24 h. Cells were stained for CD86 using BD cytofix/cytoperm fixation/permeabilization solution kit according to manufacturer’s protocol. Cells were analyzed using NovoCyte flow cytometer (ACEA Biosciences, Inc).

#### *In vivo* Assays

All animal procedures were performed under a protocol approved by the University of Chicago Institutional Animal Care and Use Committee (IACUC). 6–8 week-old C57/B6 female mice were purchased from Jackson Laboratory (JAX). 6–8 week-old C57/B6 female Trpv1^tm1Ju^ mice were purchased from JAX for TRPV1 KO experiment. All compounds were tested for endotoxin prior to use. All vaccinations were administered intramuscularly in the hind leg. Blood was collected from the submandibular vein at time points indicated.

Antigens were purchased from Invitrogen (VacciGrade Ovalbumin). VacciGrade CpG ODN 1826 was purchased from Adipogen. AddaVax^TM^ was purchased from Invivogen.

#### Vaccination

Mice were lightly anesthetized with isoflurane and injected intramuscularly in the hind leg with 50 μL containing ovalbumin (100 μg), adjuvant, inhibitor and PBS. Adjuvant doses: CpG, 50 μg. Inhibitor concentrations: Honokiol (400 μg), Capsaicin (20 μg), Withaferin A (600 μg), acetaminophen (2 mg), ibuprofen (800 μg). All vaccines contained 25 μL AddaVax^TM^ to enhance solubility.

#### Plasma Cytokine Analysis

Blood was collected from mice at time points indicated in 0.2 mL heparin coated collection tubes (VWR Scientific). Serum was isolated via centrifugation 2000 × *g* for 5 min. Supernatant was collected and stored at −80°C until use. Serum was analyzed using BD Cytometric Bead Array Mouse Inflammation cytokine kit or LEGENDplex^TM^ Mouse Inflammation Panel (Biolegend) according to manufacturer’s protocol.

#### Antibody Quantification

Mice were vaccinated with indicated formulations. Blood was collected at time points indicated in 0.2 mL heparin coated collection tubes (VWR Scientific) for plasma or uncoated tubes for serum. Plasma was isolated via centrifugation (2000 × *g*, 5 min). Serum was isolated by allowing blood to clot for 15–30 min RT and centrifuging (2000 × *g* for 10 min) at 4°C. Serum was analyzed using a quantitative anti-ovalbumin total Ig’s ELISA kit (Alpha Diagnostic International) according to the specified protocol. Data was analyzed using Graphpad Prism.

### Chemistry

#### Conditions for Suzuki Coupling

Hydroxyphenylboronic acid (20 mmol) was dissolved in 100 mL water. Appropriate iodophenol (10 mmol) and K_2_CO_3_ (40 mmol) was added followed by Pd/C (2 mol %). Solution heated to 80 C for 3 h. Solution was acidified with 1M HCl and extracted with EtOAc and washed with brine. Solvent evaporated in vacuo. Compound was purified by column chromatography.

#### Conditions for O-Allylations

Phenol (1 mmol) (Derivative **1**–**8**) was dissolved in dry acetone (5 mL) and K_2_CO_3_ (2 mmol) added. AllylBr was added dropwise and refluxed. Reaction was monitored by TLC until completion (5–12 h). Reaction mixture was cooled and volatiles were removed in vacuo. 10% NaOH was added to the mixture and extraction was performed using ethyl acetate, washed with brine and organic layers dried using MgSO_4_. Solvent was removed in vacuo affording an oily material that was purified by column chromatography to yield the O-allylated derivative.

#### Conditions for Claisen Rearrangement

O-allylated derivatives (**9**–**17**) (1 mmol) were dissolved in dry hexane (10 mL). Et_2_AlCl in dry hexane (4 mL) was added dropwise under argon. Mixture was stirred at room temperature for 2 h. The mixture was cooled on an ice bath and quenched using 2M HCl (20 mL). Extraction was performed with EtOAc, washed with brine and dried over MgSO4. Solvent was removed in vacuo affording an oily material that was purified by column chromatography to yield the C-allyl derivative.

#### Statistics and Replicates

Data is plotted and reported in the text as the mean ± s.e.m. Sample size is as indicated in biological replicates in all *in vivo* and *in vitro* experiments. The sample sizes were chosen based on preliminary experiments or literature precedent indicating that the number would be sufficient to detect significant differences in mean values should they exist. *P*-values were calculated using a one-way ANOVA and Tukey *post hoc* test. All experiments have been repeated (sometimes with minor variations due to reagents and materials) and replication was successful.

## Author’s Note

This manuscript has been released as a Pre-Print at ChemRxiv (32).

## Data Availability Statement

All datasets generated for this study are included in the article/Supplementary Material.

## Ethics Statement

The animal study was reviewed and approved by the University of Chicago IACUC.

## Author Contributions

BM and AE-K conceived of and designed the project and experiments, and wrote the manuscript. BM, YE-B, RS, MR, BC, MN, and NT performed the experiments. BM synthesized the materials. BM, NN, and AC performed the compound characterization. All authors contributed to the article and approved the submitted version.

## Conflict of Interest

BM and AE-K are inventors on a pending patent related to this work filed by the University of Chicago (no. PCT/US19/64888, filed 16 December 2019).

The remaining authors declare that the research was conducted in the absence of any commercial or financial relationships that could be construed as a potential conflict of interest.
