# Supplementary material for: Small Molecule NF-κB Inhibitors as Immune Potentiators for Enhancement of Vaccine Adjuvants
Source: Front Immunol. 2020 Sep 25;11:511513. doi: 10.3389/fimmu.2020.511513 (PMC7544742; doi:10.3389/fimmu.2020.511513)
Supplement: Supplementary file 1 [file Data_Sheet_1.pdf]

Supplementary Material

This PDF file includes:

Figs. S1 to S3

Table S1

Supplementary Materials and Methods

Supplementary References

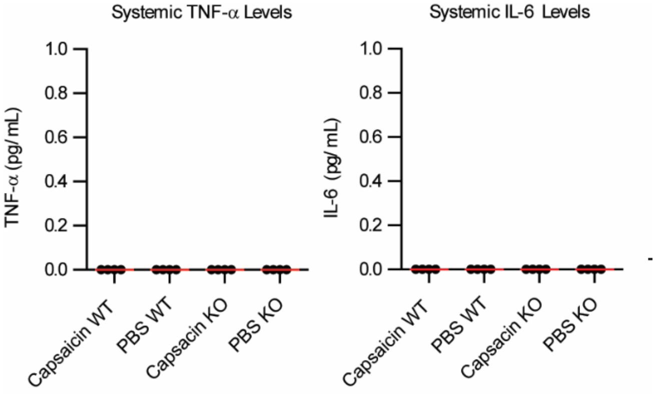

Figure S1. Capsaicin and PBS alone vaccinations in wild type and TRPV1 KO mice. Systemic TNF-α or IL-6 levels 1 h post vaccination with capsaicin or PBS in wild type (WT) mice and TRPV1 KO (KO).

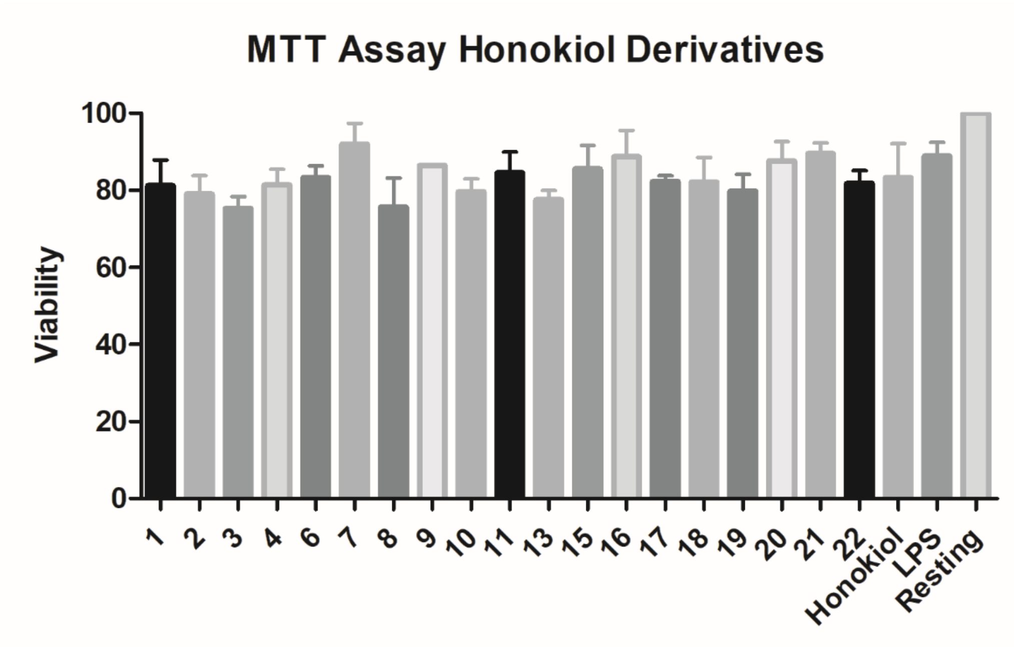

Figure S2. Cell viability of RAW macrophages treated with honokiol derivative library. MTT assay examining cell viability of RAW macrophages treated with honokiol derivatives and LPS for 24 h.

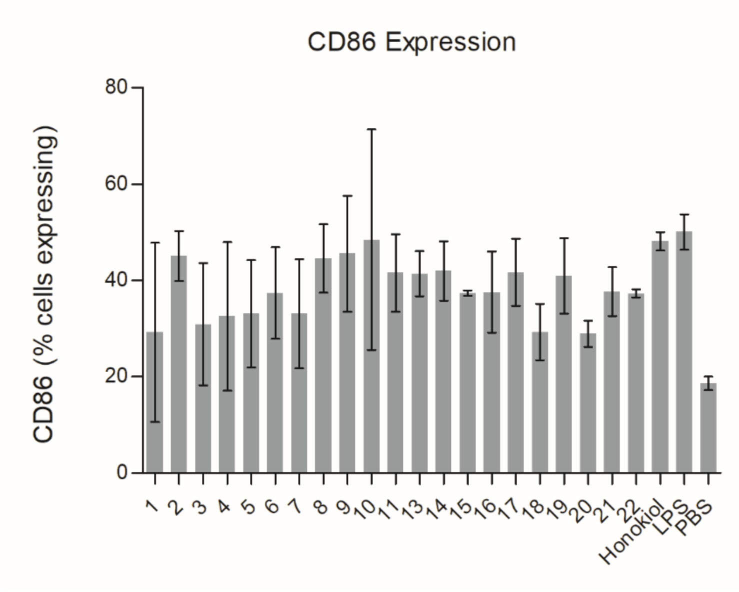

Figure S3. CD86 expression in RAW macrophages. CD86 cell surface staining of RAW macrophages treated with honokiol derivatives and LPS for 24 h.

Supplementary Table 1

Compound	Inhibition of NF-kB Pathway	Reference
Cardamonin	Inhibition of IκBα degradation and phosphorylation & inhibition of IκB kinase activation	[1]
Caffeic acid phenethyl ester (CAPE)	Inhibition of IκBα degradation and phosphorylation & inhibition of IκB kinase activation	[2]
Withaferin A (WA)	Inhibition of NEMO/IKKβ complex	[3]
Resveratrol	Inhibition of NEMO ubiquitination	[4]
Salicin	Inhibition of IKKb	[5]
5Z-7-Oxozeaenol (5-z-O)	Inhibition of TAK1	[6]
Parthenolide	Inhibition of IKK, direct inhibition of NF-kB DNA binding	[7]
Honokiol	Inhibition of IκBα phosphorylation and IκBα degradation	[8]
Capsaicin	Inhibition of IκBα phosphorylation and IκBα degradation	[9]
PDK1/Akt/Flt dual pathway inhibitor (PDK1)	Inhibition of PDK1	[10]
GYY 4137 (GYY)	Inhibition of IκBα degradation	[11]

As small molecules, these NF-kB inhibitors may impact other pathways. For example, CAPE has been identified to exert its inhibition through electrophilic addition to sulfhydryl groups. [2] Therefore, CAPE will impact many off-target proteins (although how this alters affect is still unknown). Honokiol is known to impact a variety of pathways such as: STAT3, EGFR, mTOR and caspase-mediated common pathways. [12] We caution the reader to be careful when drawing conclusions about which aspects of the NF-kB pathways are important for decreases in cytokines and immune potentiators with small molecules. NF-kB inhibitors have been extensively examined and reviewed elsewhere and we direct the reader to these reviews for more information on NF-kB inhibitors and their mode of inhibition.[13,14]

Supplementary Materials and Methods

Chemistry

Conditions for Suzuki Coupling

Hydroxyphenol boronic acid (20 mmol) was dissolved in 100 mL water. Appropriate iodophenol (10 mmol) and K_2CO_3 (40 mmol) was added followed by Pd/C (2 mol %). Solution heated to 80 C for 3h. Solution was acidified with 1M HCl and extracted with EtoAc and washed with brine. Solvent evaporated in vacuo. Compound was purified by column chromatography.

1, **2**, **3**, **8** were purchased from Sigma Aldrich.

(4): Spectral data as previously described: Schmidt, B.; Riemer, M., Journal of Organic Chemistry, 2014, 4104 – 4118.

^{1}H NMR (400 MHz, $CDCl_3$) δ 7.54 – 7.38 (m, 2H), 7.37 – 7.27 (m, 1H), 7.10 (ddd, *J* = 7.7, 1.7, 1.0 Hz, 1H), 7.08 – 6.96 (m, 1H), 6.93 – 6.85 (m, 2H), 6.82 – 6.73 (m, 1H).

HRMS (ESI) calculated for C24H18O3 [2M-H_2O]+: 354.1256, found: 354.1259.

(5): Spectral data as previously described: Reddy, B.V.S; Rao, R.N.; Reddy, N.S.S.; Somaiah, R.; Yadav, J.S.; Subramanyam, R., Tetrahedron Letters, 2014, 1049 – 1051.

^{1} H NMR (400 MHz, $CDCl_3$) δ 7.35 (d, *J* = 8.6 Hz, 2H), 7.25 – 7.18 (m, 2H), 7.03 – 6.93 (m, 4H).

HRMS (ESI) calculated for C18H19O2 [M+H]+: 267.1385, found: 267.1400.

(6): Spectral data as previously described: Moorthy, J. ; Venkatakrishnan, P.; Samanta, S., Organic and Biomolecular Chemistry, 2007, 1354 – 1357.

^{1}H NMR (400 MHz, $CDCl_3$) δ 7.30 (t, *J* = 7.9 Hz, 2H), 7.14 (ddd, *J* = 7.7, 1.7, 1.0 Hz, 2H), 7.04 (dd, *J* = 2.5, 1.6 Hz, 2H), 6.82 (ddd, *J* = 8.1, 2.6, 1.0 Hz, 2H).

HRMS (ESI) calculated for C12H9O2 [M-H]-: 185.0603, found: 185.0633.

(7): Spectral data as previously described: Sánchez-Peris, M.; Falomir, E.; Murga, J.; Carda, M.; Marco, J. Bioorganic and Medicinal Chemistry, 2016, 3108 – 3115.

^{1}H NMR (400 MHz, $CDCl_3$) δ 7.30 (t, *J* = 7.9 Hz, 1H), 7.25 – 7.16 (m, 2H), 7.00 – 6.93 (m, 3H), 6.91 (dd, *J* = 2.6, 1.6 Hz, 1H), 6.83 (ddd, *J* = 8.2, 2.6, 1.0 Hz, 1H).

HRMS (ESI) calculated for C12H9O2 [M+H]-: 185.0603, found: 185.0635.

Conditions for O-allylations

Phenol (1 mmol) (Derivative **1-8**) was dissolved in dry acetone (5 mL) and K_2CO_3 (2 mmol) added. AllylBr was added dropwise and refluxed. Reaction was monitored by TLC until completion (5-12h). Reaction mixture was cooled and volatiles were removed in vacuo. 10% NaOH was added to the

mixture and extraction was performed using ethyl acetate, washed with brine and organic layers dried using $MgSO_4$. Solvent was removed in vacuo affording an oily material that was purified by column chromatography to yield the O-allylated derivative.

(**9**): Spectral data as previously reported: Khan, A.; Komejan, S.; Patel, A.; Lombardi, C.; Lough, A.; Foucher, D., Journal of Organometallic Chemistry, 2015, 180 – 191.

^{1}H NMR (400 MHz, $CDCl_3$) δ 7.54 (ddt, *J* = 11.8, 5.2, 2.2 Hz, 4H), 7.46 – 7.37 (m, 2H), 7.34 – 7.28 (m, 1H), 7.04 – 6.96 (m, 2H), 6.09 (ddt, *J* = 17.2, 10.5, 5.3 Hz, 1H), 5.45

(dq, *J* = 17.3, 1.6 Hz, 1H), 5.31 (dq, *J* = 10.5, 1.4 Hz, 1H), 4.59 (dt, *J* = 5.3, 1.5 Hz, 2H).

HRMS (ESI) calculated for C30H32NO [2M+NH4]+: 438.2471, found: 438.2403.

(10): Spectral data as reported: Bujok, R.; Bieniek, M.; Masnyk, M.; Michrowska, A.; Sarosiek, A.;Stępowska, H.; Arlt, D.; Grela, K. J. Org. Chem. 2004, 69, 6894-6896.

^{1}H NMR (400 MHz, $CDCl_3$) δ 7.58 (dq, *J* = 2.6, 1.7 Hz, 2H), 7.48 – 7.38 (m, 2H), 7.38 –

7.27 (m, 3H), 7.09 – 7.02 (m, 1H), 6.99 (dd, *J* = 8.2, 0.8 Hz, 1H), 6.00 (ddt, *J* = 17.3, 10.6, 4.8 Hz, 1H), 5.34 (dq, *J* = 17.3, 1.7 Hz, 1H), 5.21 (dq, *J* = 10.6, 1.6 Hz, 1H), 4.55 (dt, *J* = 4.8, 1.7 Hz, 2H).

HRMS (ESI) calculated for C15H15O [M+H]+: 211.1127, found: 211.1125.

(**11**): Spectral data as previously reported: Sánchez-Peris, M., Murga, J., Falomir, E., Carda, M., & Marco, J. A. Chemical biology & drug design 2017, 577-584.

^{1}H NMR (400 MHz, $CDCl_3$) δ 7.59 (dt, *J* = 8.4, 2.5 Hz, 2H), 7.43 (dt, *J* = 6.9, 4.8 Hz,

2H), 7.34 (td, *J* = 7.8, 2.2 Hz, 2H), 7.23 – 7.18 (m, 1H), 7.16 (q, *J* = 2.3 Hz, 1H), 6.91 (dd, *J* = 8.2, 2.7 Hz, 1H), 6.09 (dddd, *J* = 15.8, 8.0, 6.6, 4.1 Hz, 1H), 5.45 (dq, *J* = 17.3, 1.6 Hz, 1H), 5.31 (dq, *J* = 10.5, 1.5 Hz, 1H), 4.65 – 4.54 (m, 2H).

HRMS (ESI) calculated for $C_{15}H_{15}O$ [M+H]+: 211.1123, found: 211.1125.

(12): Spectral data as previously reported: Schlosser, M.; Michel, D.; Croft, S. Synthesis 1996, 591-593.

^{1}H NMR (400 MHz, $CDCl_3$) δ 7.49 – 7.44 (m, 4H), 7.03 – 6.90 (m, 4H), 6.08 (ddt, *J* = 17.2, 10.6, 5.3 Hz, 2H), 5.44 (dq, *J* = 17.3, 1.6 Hz, 2H), 5.30 (dq, *J* = 10.5, 1.4 Hz, 2H),

4.57 (dt, *J* = 5.3, 1.5 Hz, 4H).

HRMS (ESI) calculated for C18H19O2 [M+H]+: 267.1394, found: 267.1389.

(13): Spectral data as previously reported: Tripathi, S.; Chan, M; Chen, C. Bioorg. Med. Chem. Lett. 2012, 22, 216-221.

^{1}H NMR (400 MHz, $CDCl_3$) δ 7.56 – 7.48 (m, 2H), 7.32 (t, *J* = 7.9 Hz, 1H), 7.14 (ddd, *J* =

7.7, 1.6, 0.9 Hz, 1H), 7.12 – 7.09 (m, 1H), 7.02 – 6.94 (m, 2H), 6.86 (ddd, *J* = 8.2, 2.6, 0.9 Hz, 1H), 6.21 – 6.02 (m, 2H), 5.44 (ddd, *J* = 17.3, 3.1, 1.5 Hz, 2H), 5.34 – 5.26 (m, 2H), 4.64 – 4.55 (m, 4H).

HRMS (ESI) calculated for C18H19O2 [M+H]+: 267.1394, found: 267.1391.

(14): Spectral data as previously reported: Reddy, B.V.S; Rao, R.N.; Reddy, N.S.S.; Somaiah, R.; Yadav, J.S.; Subramanyam, R., Tetrahedron Letters, 2014, 1049 – 1051.

^{1}H, 500 MHz): δ 7.39 (d, J = 9.0 Hz, 2H), 7.14-7.21 (m, 2H), 6.86-6.90 (m, 4H), 5.87-5.97 (m, 2H), 5.08-5.32 (m, 4H), 4.46 (d, J = 5Hz, 2H), 4.41 (d, J = 5Hz, 2H)

HRMS (ESI) calculated for $C_{18}H_{18}O_2$ [M+H]+: 266.1307, found: 266.1316.

(15): Spectral data as previously reported: Sánchez-Peris, M., Murga, J., Falomir, E., Carda, M., & Marco, J. A. Chemical biology & drug design 2017, 577-584.

^{1}H NMR (400 MHz, $CDCl_3$) δ 7.36 (t, *J* = 7.9 Hz, 2H), 7.20 (ddd, *J* = 7.6, 1.7, 1.0 Hz,

2H), 7.17 (dd, *J* = 2.6, 1.6 Hz, 2H), 6.93 (ddd, *J* = 8.2, 2.5, 1.0 Hz, 2H), 6.11 (ddt, *J* = 17.3, 10.5, 5.3 Hz, 2H), 5.47 (dq, *J* = 17.3, 1.6 Hz, 2H), 5.33 (dq, *J* = 10.5, 1.4 Hz, 2H),

4.62 (dt, *J* = 5.3, 1.6 Hz, 4H).

HRMS (ESI) calculated for $C_{18}H_{18}O_2$ [M+H]+: 267.1385, found: 267.1386.

(16): Spectral data as previously reported: Sánchez-Peris, M., Murga, J., Falomir, E., Carda, M., & Marco, J. A. Chemical biology & drug design 2017, 577-584.

^{1}H NMR (400 MHz, $CDCl_3$) δ 7.38 – 7.33 (m, 1H), 7.33 – 7.27 (m, 2H), 7.16 (ddd, *J* =

5.3, 2.7, 1.4 Hz, 2H), 7.04 (td, *J* = 7.5, 1.1 Hz, 1H), 6.98 (dd, *J* = 8.3, 1.1 Hz, 1H), 6.90 (ddd, *J* = 8.2, 2.6, 1.1 Hz, 1H), 6.17 – 5.92 (m, 2H), 5.39 (ddq, *J* = 35.8, 17.3, 1.7 Hz, 2H), 5.26 (ddq, *J* = 30.6, 10.6, 1.5 Hz, 2H), 4.56 (ddt, *J* = 10.4, 4.9, 1.6 Hz, 4H).

HRMS (ESI) calculated for C18H19O2 [M+H]+: 267.1394, found: 267.1395.

(17): Spectral data as previously reported: Sánchez-Peris, M., Murga, J., Falomir, E., Carda, M., & Marco, J. A. Chemical biology & drug design 2017, 577-584.

^{1}H NMR (400 MHz, $CDCl_3$) δ 7.29 (dtd, *J* = 8.2, 7.2, 3.8 Hz, 4H), 7.09 – 6.99 (m, 2H),

6.95 (d, *J* = 8.2 Hz, 2H), 5.92 (ddt, *J* = 17.3, 10.6, 4.8 Hz, 2H), 5.22 (ddd, *J* = 17.3, 3.5, 1.8 Hz, 4H), 5.13 (ddd, *J* = 10.6, 3.2, 1.6 Hz, 4H), 4.51 (dt, *J* = 4.6, 1.7 Hz, 4H).

HRMS (ESI) calculated for $C_{18}H_{18}O_2$ [M+H]+: 267.1385, found: 267.1380.

Conditions for Claisen rearrangement

O-allylated derivatives (**9**-**17**) (1 mmol) were dissolved in dry hexane (10 mL). Et_2AlCl in dry hexane (4 mL) was added dropwise under argon. Mixture was stirred at room temperature for 2h. The mixture was cooled on an ice bath and quenched using 2M HCl (20 mL). Extraction was performed with EtOAc, washed with brine and dried over MgSO4. Solvent was removed in vacuo affording an oily material that was purified by column chromatography to yield the C-allyl derivative.

(18): Spectral data as previously reported: Sánchez-Peris, M., Murga, J., Falomir, E., Carda, M., & Marco, J. A. Chemical biology & drug design 2017, 577-584.

^{1}H NMR (400 MHz, $CDCl_3$) δ 7.60 – 7.51 (m, 2H), 7.47 – 7.34 (m, 5H), 7.34 – 7.27 (m, 1H), 6.89 (d, *J* = 8.1 Hz, 1H), 6.06 (ddt, *J* = 17.2, 10.1, 6.4 Hz, 1H), 5.26 – 5.16 (m, 2H), 3.49 – 3.46 (m, 2H).

HRMS (ESI) calculated for C18H19O2 [M+H]+: 267.1394, found: 267.1381.

(19): Spectral data as previously reported: Sánchez-Peris, M., Murga, J., Falomir, E., Carda, M., & Marco, J. A. Chemical biology & drug design 2017, 577-584.

^{1}H NMR (400 MHz, $CDCl_3$) δ 7.54 – 7.44 (m, 5H), 7.43 – 7.35 (m, 1H), 7.14 (ddd, *J* =

9.3, 4.6, 1.1 Hz, 2H), 6.95 (t, *J* = 7.5 Hz, 1H), 6.07 (ddt, *J* = 16.6, 10.0, 6.6 Hz, 1H), 5.14 (qdd, *J* = 3.2, 2.6, 1.5 Hz, 2H), 3.48 (d, *J* = 6.6 Hz, 2H).

HRMS (ESI) calculated for C15H15O [2M+H]+: 421.2168, found: 421.2173.

(20): Spectral data as previously reported: Eisai R&D Management Co., Ltd. - EP1847535A1, **2007**.

^{1}H NMR (400 MHz, $CDCl_3$) δ 7.60 – 7.54 (m, 1H), 7.48 – 7.28 (m, 4H), 7.23 – 7.16 (m,

1H), 7.14 (dd, *J* = 7.8, 1.8 Hz, 0H), 7.07 (d, *J* = 1.7 Hz, 0H), 6.92 – 6.86 (m, 1H), 6.17 –

5.91 (m, 1H), 5.26 – 5.06 (m, 2H), 3.46 (dt, *J* = 6.4, 1.7 Hz, 1H), 3.35 (dt, *J* = 5.6, 1.9 Hz, 1H).

HRMS (ESI) calculated for $C_{15}H_{15}O$ [M+H]+: 211.1123, found: 211.1125.

(21): Spectral data as previously reported: M.-Y. Chang, S.-Y. Lin, C.-K. Chan, Tetrahedron 2013, 69, 2933-2940.

^{1}H NMR (400 MHz, $CDCl_3$) δ 7.38 – 7.22 (m, 4H), 6.87 (t, *J* = 10.0 Hz, 2H), 6.06 (ddt, *J* = 16.5, 10.1, 6.3 Hz, 2H), 5.28 – 5.12 (m, 4H), 3.47 (d, *J* = 6.3 Hz, 4H).

HRMS (ESI) calculated for C18H19O2 [M+H]+: 267.1394, found: 267.1408.

(22): Spectral data as previously reported: Sánchez-Peris, M., Murga, J., Falomir, E., Carda, M., & Marco, J. A. Chemical biology & drug design 2017, 577-584.

^{1}H NMR (400 MHz, $CDCl_3$) δ 7.21 – 7.00 (m, 3H), 6.91 – 6.66 (m, 3H), 6.16 – 5.78 (m, 2H), 5.25 – 4.88 (m, 4H), 3.54 – 3.32 (m, 4H).

HRMS (ESI) calculated for $C_{18}H_{18}O_2$ [M+H]+: 267.1385, found: 267.1390.

Supplementary References

(1) Israf, D. A., Khaizurin, T. A., Syahida, A., Lajis, N. H., & Khozirah, S. Cardamonin inhibits COX and iNOS expression via inhibition of p65NF-κB nuclear translocation and Iκ-B phosphorylation in RAW 264.7 macrophage cells. *Molecular immunology*, *44*(5), 673-679 (2007).

(2) Grover, A., Shandilya, A., Punetha, A., Bisaria, V. S., & Sundar, D. Inhibition of the NEMO/IKKβ association complex formation, a novel mechanism associated with the NF-κB activation suppression by Withania somnifera's key metabolite withaferin A. *BMC genomics*, 11(4), S25 (2010).

(3) Ren, Z., Wang, L., Cui, J., Huoc, Z., Xue, J., Cui, H., Mao, Q. Yang, R. & Yang, R. Resveratrol inhibits NF-κB signaling through suppression of p65 and IB kinase activities. *Die Pharmazie-An International Journal of Pharmaceutical Sciences*, *68*(8), 689-694 (2013).

(4) Kopp, E., & Ghosh, S. Inhibition of NF-kappa B by sodium salicylate and aspirin. *Science*, *265*(5174), 956-959 (1994).

(5) Wu, J., Powell, F., Larsen, N. A., Lai, Z., Byth, K. F., Read, J., Rong-Fang, G. Roth, M., Toader, D. Saeh, J.C. & Chen, H. Mechanism and in vitro pharmacology of TAK1 inhibition by (5 Z)-7-oxozeaenol. *ACS chemical biology*, *8*(3), 643-650 (2013).

(6) Hehner, S. P., Heinrich, M., Bork, P. M., Vogt, M., Ratter, F., Lehmann, V., Schulze-Osthoff, K., Droge, W & Schmitz, M. L. Sesquiterpene lactones specifically inhibit activation of NF-κB by preventing the degradation of IκB-α and IκB-β. *Journal of Biological Chemistry*, *273*(3), 1288-1297 (1998).

(7) Qiu, L., Xu, R., Wang, S., Li, S., Sheng, H., Wu, J., & Qu, Y. Honokiol ameliorates endothelial dysfunction through suppression of PTX3 expression, a key mediator of IKK/IκB/NF-κB, in atherosclerotic cell model. *Experimental & molecular medicine*, *47*(7), e171-e171 (2015).

(8) Kim, C. S., Kawada, T., Kim, B. S., Han, I. S., Choe, S. Y., Kurata, T., & Yu, R. Capsaicin exhibits anti-inflammatory property by inhibiting IkB-a degradation in LPS-stimulated peritoneal macrophages. *Cellular signalling*, *15*(3), 299-306 (2003).

(9) Zeng, Z., Samudio, I.J., Zhang, W., Estrov, Z., Pelicano, H., Harris, D., Frolova, O., Hail, N., Chen, W., Kornblau, S.M. and Huang, P. Simultaneous inhibition of PDK1/AKT and Fms-like tyrosine kinase 3 signaling by a small-molecule KP372-1 induces mitochondrial dysfunction and apoptosis in acute myelogenous leukemia. *Cancer research*, *66*(7), pp.3737-3746 (2006).

(10) Wu, Z., Peng, H., Du, Q., Lin, W. and Liu, Y. GYY4137, a hydrogen sulfide-releasing molecule, inhibits the inflammatory response by suppressing the activation of nuclear factor-kappa B and mitogen-activated protein kinases in Coxsackie virus B3-infected rat cardiomyocytes. *Molecular medicine reports*, *11*(3), pp.1837-1844 (2015).

(11) Kumar, A.; Kumar Singh, U.; Chaudhary, A. Honokiol Analogs: A Novel Class of Anticancer Agents Targeting Cell Signaling Pathways and Other Bioactivities. *Future Medicinal Chemistry*, *5* (7), 809–829 (2013).

(12) Gilmore, T. D.; Herscovitch, M. Inhibitors of NF-KB Signaling: 785 and Counting. *Oncogene 25*, 6887 (2005).

(13) Arepalli, S. K., Choi, M., Jung, J. K., & Lee, H. Novel NF-κB inhibitors: A patent review (2011–2014). *Expert opinion on therapeutic patents*, *25*(3), 319-334 (2015).
